# Construction and Evaluation of the Immunogenicity and Protective Efficacy of Recombinant Replication-Deficient Human Adenovirus-5 Expressing Genotype VII Newcastle Disease Virus F Protein and Infectious Bursal Disease Virus VP2 Protein

**DOI:** 10.3390/vaccines11061051

**Published:** 2023-05-31

**Authors:** Ting Xu, Ting Xiong, Wenting Xie, Jing Wu, Xiao Liu, Guimin Li, Yadi Lv, Linyu Li, Zekun Yang, Han Wang, Dingxiang Liu, Ruiai Chen

**Affiliations:** 1 Zhaoqing Branch of Guangdong Laboratory of Lingnan Modern Agricultural Science and Technology, Zhaoqing 526238, China; 15662032466@163.com (T.X.); bearvet@163.com (T.X.); m13517313047@163.com (L.L.); 2 College of Veterinary Medicine, South China Agricultural University, Guangzhou 510640, China; xwtseventeen2023@163.com (W.X.); 1036946343@stu.scau.edu.cn (J.W.); liuxiaof152@163.com (X.L.); m13189167745@163.com (G.L.); 15716327913@163.com (Y.L.); zekunyang961224@163.com (Z.Y.); wanghanpaprika@163.com (H.W.); 3 Integrative Microbiology Research Centre, South China Agricultural University, Guangzhou 510642, China; 4 Key Laboratory of Biotechnology and Bioproducts Development for Animal Epidemic Prevention, Ministry of Agriculture and Rural Affairs, Zhaoqing 526238, China; 5 Guangdong Enterprise Key Laboratory of Biotechnology R&D of Veterinary Biologics, Zhaoqing 526238, China; 6 Zhaoqing Dahuanong Biology Medicine Co., Ltd., Zhaoqing 526238, China

**Keywords:** NDV F protein, IBDV VP2 protein, rHAdV-5-based vaccine candidates, humoral immune response, cellular immune response

## Abstract

Newcastle disease (ND) and infectious bursal disease (IBD) are two key infectious diseases that significantly threaten the health of the poultry industry. Although existing vaccinations can effectively prevent and treat these two diseases through multiple immunizations, frequent immunization stresses significantly impact chicken growth. In this study, three recombinant adenoviruses, rAd5-F expressing the NDV (genotype VII) F protein, rAd5-VP2 expressing the IBDV VP2 protein, and rAd5-VP2-F2A-F co-expressing F and VP2 proteins, were constructed using the AdEasy system. The F and VP2 genes of the recombinant adenoviruses could be transcribed and expressed normally in HEK293A cells as verified by RT-PCR and Western blot. The three recombinant viruses were shown to have similar growth kinetics as rAd5-EGFP. Compared with the PBS and rAd5-EGFP groups, SPF chickens immunized with recombinant adenoviruses produced higher antibody levels, more significant lymphocyte proliferation, and significantly higher CD4+/CD3+ and CD8+/CD3+ cells in peripheral blood. The survival rate of SPF chickens immunized with rAd5-F and rAd5-VP2-F2A-F after the challenge with DHN3 was 100%, and 86% of SPF chickens showed no viral shedding at 7 dpc. The survival rate of SPF chickens immunized with rAd5-VP2 and rAd5-VP2-F2A-F after the challenge with BC6/85 was 86%. rAd5-VP2 and rAd5-VP2-F2A-F significantly inhibited bursal atrophy and pathological changes compared to the rAd5-EGFP and PBS groups. This study provides evidence that these recombinant adenoviruses have the potential to be developed into safe and effective vaccine candidates for the prevention and control of ND and IBD.

## 1. Introduction

Newcastle disease virus (NDV) infects all bird species worldwide, causing acute gastrointestinal and respiratory symptoms, including dysentery, high fever, respiratory distress, and neurological disorders. In addition, variations in mortality and morbidity rates were observed between bird species and virus strains [1]. NDV belongs to the genus *Avulavirus*, subfamily *Paramyxovirinae*, family *Paramyxoviridae* [2], and contains a single-stranded, non-segmented, negative-sense RNA genome [3]. The viral genome can direct the transcription of mRNAs that encode for six structural proteins, namely fusion glycoprotein (F), phosphoprotein (P), matrix protein (M), hemagglutinin-neuraminidase (HN) glycoprotein, nucleocapsid protein (NP) and large polymerase protein (L), in addition to two non-structural proteins V and W produced by RNA editing during the P gene transcription [4]. The HN and F proteins are surface glycoproteins, the main virulence factors, and immunoprotective antigens [5,6]. The current genetic variation of NDV is mainly focused on the F gene, which is also the primary basis for genotype classification. The F protein exists as a trimer on the surface of viral particles. Its main biological function is to mediate the fusion of the viral envelope with the membranes of the host cell [7], thus allowing the virus to invade the host cell. Therefore, the F gene is the main target for the development of a subunit vaccine against NDV infection.

Infectious bursal disease virus (IBDV), belonging to the genus *Avian birnavirus* of the family *Birnaviridae* [8], mainly infects chicks from 3–6 weeks of age and causes an immunosuppressive infectious disease [9]. The virus mainly attacks the bursa of Fabricius, inducing necrosis and collapse of B lymphocytes, thus causing permanent damage to immune organs and inducing other diseases in poultry [10]. There is currently no effective cure for the disease, which has resulted in significant economic losses for the poultry sector [11]. IBDV is a non-enveloped virus enclosing two double-stranded RNA segments (A and B). Fragment A contains two open reading frames (ORFs): the larger ORF encodes a precursor protein pVP2, a capsid scaffolding protein VP3, and a viral serine protease VP4 (NH3-pVP2-VP4-VP3-COOH). Subsequently, pVP2 is processed into the mature capsid protein VP2. The smaller ORF of fragment A encodes the VP5 protein. The ORF of fragment B encodes only the protein VP1 [10,12]. The VP2 gene is the main virulence gene, and this protein accounts for approximately 51% of the total proteins in the viral particles [13]. As the primary protective antigen, the VP2 protein contains multiple regions associated with antigenicity. The immunoprotective efficacy of IBDV vaccines was shown to be directly related to the level of characteristic antibodies against VP2 [14]. In addition, several viral-neutralizing antigenic epitopes recognized by neutralizing antibodies were also identified in this protein [15,16]. Therefore, the VP2 gene is an important target gene in the study of genetically engineered IBDV vaccines.

Vaccination is an important measure to control NDV and IBDV infections. With the continuous development of molecular biology, viral vector vaccines have excellent application prospects. Replication-deficient human adenovirus serotype-5 (HAdV-5), a commonly used vaccine vector, has high stability, large packaging capacity, good safety record, broad host range, and high expression efficiency of heterogeneous antigens due to effective proliferation [17,18]. These traits would make it an ideal vector for the development of bi- and multivalent animal vaccines. Its application in the development of animal vaccines can also effectively avoid the interference of maternal antibodies [19]. This study developed three recombinant adenoviruses, rAd5-F, rAd5-VP2, and rAd5-VP2-F2A-F, using HAdV-5 as a vector. In rAd5-VP2-F2A-F, the F2A sequence (amino acid sequence: NFDLLKLAGDVESNPGP) derived from the foot-and-mouth disease virus (FMDV) was used to link F and VP2 genes in tandem. Using the 2A peptide segment, simultaneous expression of the two proteins would be achieved. After analyzing their genetic stability, growth, reproduction properties, and titers, three recombinant adenoviruses were purified to immunize SPF chickens, demonstrating effective antibody production and conferring partial protection.

## 2. Materials and Methods

The animal study was reviewed and approved by the Animal Experiments Committee of Zhaoqing Da Huanong Biopharmaceutical Co., Ltd. (Zhaoqing, China). Experiments on SPF chickens were conducted strictly with the Guide for the Care of Laboratory Animals and the experimental protocol approved by the Animal Welfare and Ethics Review Committee of South China Agricultural University.

### 2.1. Plasmids, Cells, SPF Chickens, SPF Chicken Embryos, and Viruses

The shuttle plasmid pAdTrack-GOI was provided by the School of Laboratory Medicine and Biotechnology, Southern Medical University (Guangzhou, China).

HEK293A cells (Procell CL-0003) were kindly provided by Procell Life Science & Technology Co., Ltd., and cultured in DMEM (Gibco, Grand Island, NY, USA) supplemented with 10% FBS (Gibco, USA) and 1% streptomycin-penicillin (Gibco, Grand Island, NY, USA) and incubated at 37 °C, 5% CO_2_.

Three-day-old white Leghorn SPF chicks and embryos (9-day-old) were obtained from the SPF Experimental Animal Center of Xinxing Dahua Agricultural, Poultry and Egg Co., Ltd. (Xinxing, China), with approved number: SCXK (Guangzhou, China) 2018-0019. The SPF chicks were raised in individually ventilated cages within the SPF animal house of Zhaoqing Dahuanong Biology Medicine Co., Ltd. (Zhaoqing, China).

The NDV strain DHN3 was isolated from a poultry farm in Guangdong Province, China (GenBank: MT447874.1), and the NDV attenuated strain rDHN3-mF was developed as previously described [20]. IBDV standard strain BC6/85 was provided by the China Institute of Veterinary Drug Control (No. CVCC AV7). The HVT-VP2 vector vaccine was purchased from Boehringer Ingelheim, Ingelheim am Rhein, Germany.

### 2.2. Cloning of NDV F and IBDV VP2 Genes

Total RNA from DHN3-infected cells was extracted by the Trizol method and reverse-transcribed into cDNA molecules. The F gene was amplified using primers F-F and F-R (Table 1, all primers used were synthesized by Suzhou Genewiz Biotechnology Co., Ltd., Suzhou, China), and the product was cloned into pMD19-T, giving rise to pMD19T-F. The IBDV VP2 gene (GenBank: EU595672.1) was synthesized by Suzhou Genewiz Biotechnology Co., Ltd. and cloned into pUC57, yielding pUC57-VP2.

### 2.3. Construction of Recombinant Replication-Defective Adenoviruses

Plasmids pMD19T-F and pUC57-VP2 were used as templates for PCR amplification with the designed homologous recombinant primers (Table 1), and the products were purified and used as inserted fragments for seamless cloning. For the VP2-F2A-F fusion construct, partial F2A sequences were added to the reverse primer to amplify the VP2 gene and the forward primer to amplify the F gene (Table 1). The VP2-F2A-F gene was synthesized by overlapping extension PCR (SOE PCR). The shuttle plasmid pAdTrack-GOI was digested with *Sal* I and *Xho* I and checked on 0.8% agarose gel for complete linearization. The linearized product was extracted by gel extraction and used as the vector for seamless cloning. The recombinant products were transformed into *E. coli* Trans10 competent cells (TransGen Biotech Ltd., Beijing, China) and recombinant plasmids were correctly identified and named pAdTrack-F, pAdTrack-VP2, and pAdTrack-VP2-F2A-F, respectively.

pAdTrack-F, pAdTrack-VP2, pAdTrack-VP2-F2A-F, and pAdTrack-GOI were linearized with *Pme* I to expose the homologous arms of the adenovirus backbone pAdEasy-1, and the digested products were checked by 0.8% agarose gel electrophoresis for complete linearization and gel extraction. Approximately 10 ng of the linearized products were transformed into BJ5183-AD-1 chemically competent cells (Shanghai Weidi Biotechnology Co., Ltd., Shanghai, China) for cloning the target genes into the adenovirus backbone plasmid by intra-bacterial homologous recombination. Small colonies were selected to expand the culture, and the correctly identified adenovirus plasmids were named pAd-F, pAd-VP2, pAd-VP2-F2A-F, and pAd-EGFP, respectively.

After linearization of these plasmids separately with *Pac* I and precipitation with ethanol, 2 μg of fully linearized products each was transfected into HEK293A cells with PEI transfection reagent (Polysciences, Inc., Warrington, PA, USA) and incubated at 37 °C, 5% CO_2_ for 7–10 days. Daily observation for cytopathic effects (CPE), such as cell rounding, bead-like, and shedding under the light microscope, and cluster fluorescence under the fluorescence microscope, was taken during this period. Diseased cells were repeatedly frozen/thawed at −80 °C and 37 °C three times, and the supernatants were collected after centrifugation as the P0 generation of the recombinant adenoviruses. Recombinant adenoviruses propagated in HEK293A for four consecutive generations, and their titers were determined.

### 2.4. Analysis of Target Gene Transcription

RT-PCR was used to detect the mRNA transcription status of the target gene in HEK293 cells to check if the recombinant adenovirus was successfully constructed. HEK293A cells were infected with the recombinant adenoviruses until typical CPE occurred, lysed with 3 freeze-thaw cycles at −80 °C and 37 °C, centrifuged, and 200 μL of supernatants were used for DNA/RNA extraction with AxyPrep Body Fluid Virus DNA/RNA Small Volume Kit (Axygen, Union City, CA, USA). The extracted RNAs (5 μL) were reverse-transcribed into cDNA with reverse transcription reagents (Takara, Kusatsu, Japan) in the presence of DNAase to remove the genomic DNA. The reverse transcription reaction conditions were 25 °C for 5 min, 37 °C for 45 min, and 85 °C for 5 s, using specific primers (Table 1).

### 2.5. Western Blot

HEK293A cells infected with the 4th generation of recombinant adenovirus were lysed with 2 × loading dye, scraped off with a cell scraper, and transferred to a fresh tube. After adding DTT and mixing by pipetting, the mixture was heated at 98 °C for 10 min, and 15 μL of protein samples were subjected to SDS-PAGE electrophoresis at 80 V for 20 min and 120 V for 1 h and transferred to nitrocellulose membranes (Merck Millipore Inc., Burlington, MA, USA) at 80 V for 2 h. The membranes were blocked with TBST containing 5% fat-free milk (Beyotime Biotechnology, Shanghai, China) for 1.5 h, incubated with anti-flag mouse monoclonal antibody (1:5000) and VP2 mouse monoclonal antibody (1:2000), respectively, as primary antibodies, and goat anti-mouse IgG (H+L) HRP conjugate (TransGen Biotech, Beijing, China) as the secondary antibody. An ultra-sensitive ECL chemiluminescent chromogenic solution was added to a multifunctional molecular imaging system to capture images.

### 2.6. Growth Curve Plotting and Purification of Recombinant Adenoviruses

Cells were seeded in 24-well plates 24 h before the determination of virus titer, allowed to grow to approximately 70%, infected with recombinant adenoviruses, and harvested at 12, 24, 36, 48, 60, 72, 84, 96, and 108 h, respectively, and stored at −80 °C after freeze-thawing three times. The virus solutions were 10-fold serially diluted (10^−1^–10^−6^), cells were infected for 48 h, and the viral titer was measured using the Adeno-X™ Rapid Titer Kit (Takara, Japan), while cells with DMEM containing 2% FBS only were used as a blank control group. After adding DAB for color development, cells infected with a virus dilution containing 5 to 50 brown or black cells per field were considered positive. The titer was calculated according to the formula: [(infected cells/field) × (fields/well)]/[volume virus (mL) × (dilution factor)]. Negative control cells were not stained.

The highest titers of the four recombinant adenoviruses were determined, and the 4th generation of each recombinant adenovirus was used to infect six T75 flasks of HEK293A cells. When significant CPE was observed, supernatants were harvested as described above, recombinant adenoviruses were purified using the Adenovirus Bulk Purification Kit (Biomiga, San Diego, CA, USA), and titers were measured with the Adeno-X™ Rapid Titer Kit (Takara, Japan).

### 2.7. Immunization with Recombinant Adenoviruses

Eighty-four SPF chicks (1-day-old) were divided into eight groups (Table 2) and kept in isolators for two days before immunization. For comparison purposes, one group of chicks was immunized with the HVT-VP2 vector vaccines (Boehringer Ingelheim, Germany) administered subcutaneously, and another group with the purified rDHN3-mF [20] via the nasal-ocular route. The remaining SPF chicks were divided into six groups and were injected intramuscularly with rAd5-F, rAd5-VP2-F2A-F, rAd-5-VP2, rAd5-EGFP, PBS, and without injection, respectively.

### 2.8. Detection of Serum Antibodies and Cellular Immunity

Five SPF chickens were randomly selected from each group at weekly intervals for venous blood collection at the root of the wings after immunization; specific antibody levels in the serum samples were detected by enzyme-linked immunosorbent assay (ELISA) using the IBDV antibody test kit from IDEXX, USA, and the NDV antibody test kit from Biochek, Reeuwijk, The Netherlands.

At 21 days post-immunization (dpi), peripheral blood lymphocytes were isolated from blood samples collected from the inferior wing vein of three SPF chickens in each group using the poultry peripheral blood lymphocyte isolation solution kit (Solarbio, Beijing, China). Concanavalin A (ConA) (InvivoGen, France) was used as a positive stimulus, and inactivated NDV and IBDV were used to stimulate cells from the corresponding groups. Cell-free wells with RPMI 1640 medium (Sigma, Santa Fe, NM, USA) containing 10% FBS were used as blank controls, and cells without stimulation were used as the negative control. The absorbance of peripheral blood lymphocytes at 450 nm was measured using CCK-8 (Dojindo, Japan). The proliferation stimulation index (SI) of lymphocytes was calculated as follows: SI = (mean of OD experimental group—mean of OD blank group)/(mean of OD negative control group—mean of OD blank group).

Three SPF chickens were randomly selected from each group at 28 dpi, and peripheral blood lymphocytes were isolated. The T-lymphocyte subpopulations were analyzed by flow cytometry (Beckman Coulter, Carlsbad, CA, USA) with the following antibodies: mouse anti-chicken CD3, mouse anti-chicken CD4, and mouse anti-chicken CD8α (Southern Biotech, Birmingham, AL, USA).

### 2.9. Virus Challenge

Five groups of chicks (seven chicks per group) immunized with rDHN3-mF, rAd5-F, rAd5-VP2-F2A-F, rAd5-EGFP, and PBS, respectively, were infected with 10^5^ EID_50_/0.1 mL per chick of DHN3, with the seven SPF chickens in the control group unchallenged. Daily mental status, diet, and mortality observations were conducted after the challenge and counted. Samples of oropharyngeal and cloacal swabs were collected at 3-, 5- and 7- days post-challenge (dpc), respectively, and inoculated into 9–11 days old SPF chicken embryos (three chicken embryos per sample). Chicken embryos that died within 24 h were discarded, and the remaining surviving embryos were incubated until 48 h. The allantoic fluids were then collected for the hemagglutination (HA) test to determine whether the swab contained NDV.

Chicks immunized with HVT-VP2 vector vaccines, rAd5-VP2, rAd-VP2-F2A-F, rAd5-EGFP, and PBS, respectively, were infected with 50 MID/0.2 mL per bird of IBDV BC6/85, with the seven chicks in the control group unchallenged. Daily observation of the infected chicks’ mental status, diet, and water consumption after the challenge and the mortality rate was counted. All chickens were euthanized at 7 dpc for the pathological autopsy and examination of lesions in all organs. To calculate the bursa of Fabricius and the body weight ratio of individual chickens and chickens in different groups, the size of the bursa of Fabricius of each chicken was counted (BF/BW and BBIX) as follows: BF/BW = [weight of the Bursa of Fabricius(g)/Body weight(g)] × 1000; and BBIX = (BF/BW of the experimental group)/(the mean value of BF/BW in the control group) [21]. Suppose BBIX < 0.7, the Fabricius bursa was judged as atrophy. Finally, the histopathological changes of the Fabricius bursa were compared in different groups.

### 2.10. Statistical Analysis

The GraphPad Prism8 software was used for data analysis in this trial. The one-way analysis of variance (ANOVA) method was used to statistically analyze the differences between the indicated samples and the respective control samples. Significance levels were presented by *p*-values (ns—non-significant, *p* > 0.05; * *p* < 0.05; ** *p* < 0.01; *** *p* < 0.001; **** *p* < 0.0001).

## 3. Results

### 3.1. Construction and Packaging of Recombinant Adenoviruses

**Figure 1:**
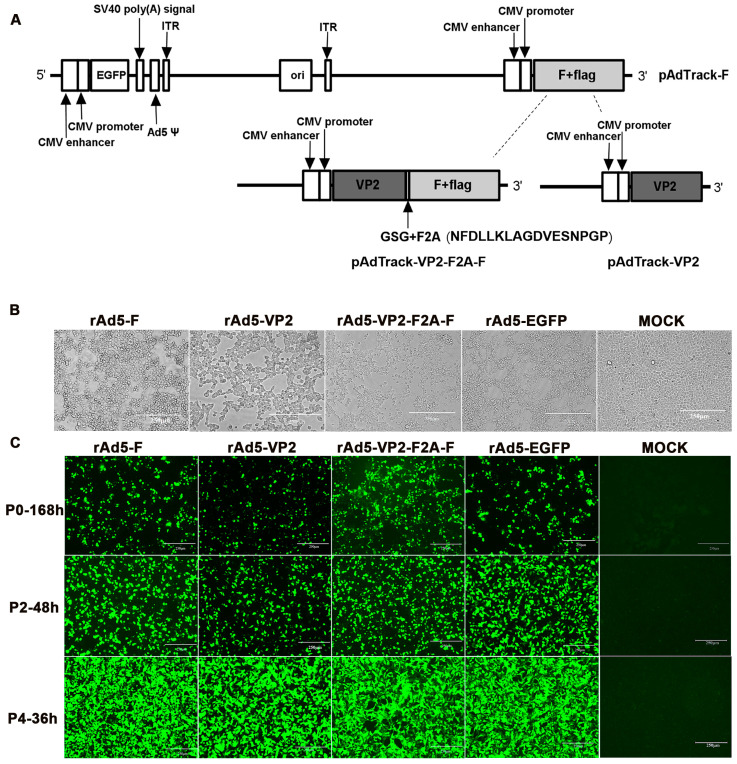
Construction and packaging of recombinant adenovirus: (**A**) Diagrams of the shuttle plasmid expression system. The Kozak sequence added upstream of the target gene, the GSG sequence added upstream of the F2A sequence, and the EGFP sequence added to the system are indicated; (**B**) CPE of recombinant adenovirus-infected HEK293A cells (200×); (**C**) Green fluorescence plot of HEK293A cells infected with different recombinant adenoviruses (200×).

Three shuttle plasmids, pAdTrack-F, pAdTrack-VP2, and pAdTrack-VP2-F2A-F, expressing NDV F, IBDV VP2, and VP2-F2A-F fusion proteins, respectively, were constructed (Figure 1A). The KOZAK consensus sequence was added at the beginning of each target gene to improve translation efficiency. In the VP2-F2A-F fusion construct, the formation of normal peptide bonds between glycine and proline in the F2A peptide is impaired by a ribosomal skip mechanism [22], resulting in the separation of the VP2 and F proteins. Adding GSG upstream of the F2A peptide would increase this cleavage efficiency and promote the expression efficiency of the target genes [23]. These recombinant shuttle plasmids were generated by intra-bacterial homologous recombination in BJ5183-AD-1 chemically competent cells that contain the pAdEasy-1 adenoviral backbone plasmid. Transfection of these recombinant adenoviral plasmids digested with *Pac* I into HEK293A cells resulted in the package of recombinant adenoviruses. HEK293A cells infected by successfully packaged recombinant adenoviruses produced obvious CPE (Figure 1B) and green fluorescence (Figure 1C). By increasing the passage of the recombinant viruses, more intense fluorescence was observed (Figure 1C).

### 3.2. Identification and Growth Curves of Recombinant Adenoviruses

**Figure 2:**
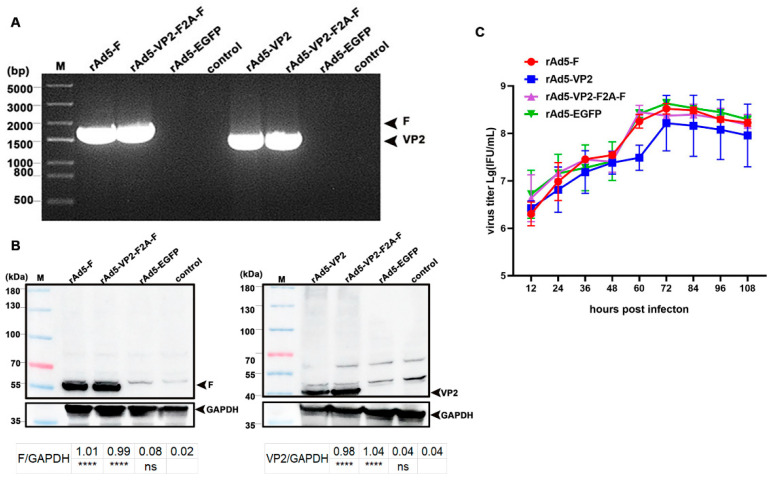
Identification and growth curves of recombinant adenoviruses: (**A**) RT-PCR analysis of transcription of the target genes; (**B**) Western blot analysis of the expression of target proteins. (ns—non-significant; **** *p* < 0.0001); (**C**) The growth curves of four recombinant adenoviruses. The growth trends of the four recombinant adenoviruses were similar.

Validation of the transcription of the 4th generation of recombinant adenoviruses expressing the target genes was performed by RT-PCR, amplifying a 1662 bp F gene fragment from rAd5-F and rAd5-VP2-F2A-F and a 1356 bp VP2 gene fragment from rAd5-VP2 and rAd5-VP2-F2A-F, respectively (Figure 2A). Further analysis by Western blot detected the 56 kDa F protein from cells infected with rAd5-F and rAd5-VP2-F2A-F and the 41 kDa VP2 protein from cells infected with rAd5-VP2 and rAd5-VP2-F2A-F (Figure 2B). None of these PCR fragments and protein bands was detected in cells infected with rAd5-EGFP and in uninfected 293A cells (Figure 2A,B).

The growth curves showed similar growth trends for the four recombinant adenoviruses, with an average titer of approximately 10^8^ IFU/mL (Figure 2C). rAd5-VP2-F2A-F reached the peak titer at 60 h and the other three at 72 h (Figure 2C). The viral titers decreased slightly afterward (Figure 2C). Therefore, the recombinant viruses were amplified and purified in large quantities for subsequent immunization of chicks. The titers of these purified recombinant viruses were as follows: rAd5-F = 2.28 × 10^9^ IFU/mL, rAd5-VP2 = 1.93 × 10^9^ IFU/mL, rAd5-VP2 = 3.51 × 10^9^ IFU/mL, rAd5-EGFP = 1.58 × 10^9^ IFU/mL.

### 3.3. Immunization with Recombinant Adenoviruses and Comparative Analysis of Serum Antibody Titers in Immunized Chickens

After injection, the SPF chicks showed no adverse clinical signs. They had a normal diet, were watered regularly, and defecated normally. This indicates that the recombinant adenovirus is safe.

**Figure 3:**
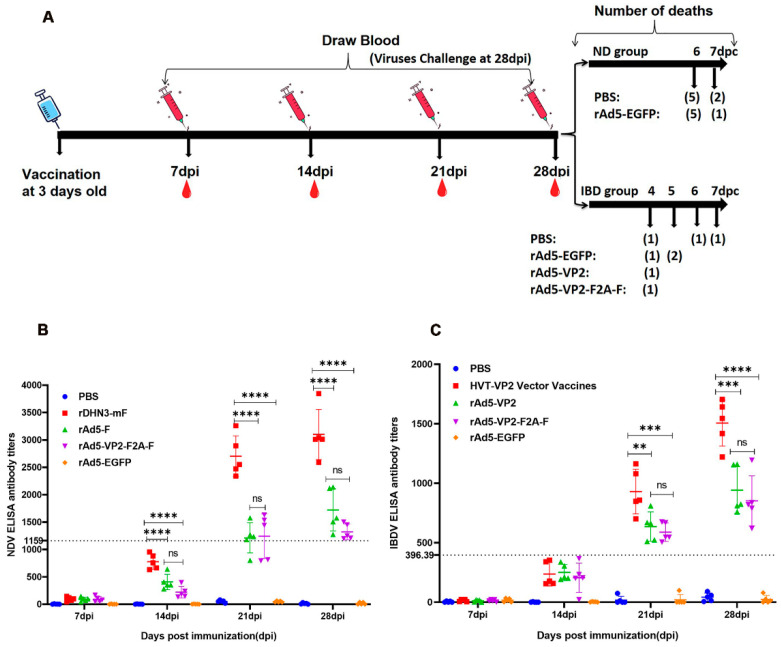
Immunization scheme and analysis of antibody levels: (**A**) Immunization and exsanguination protocol. After immunizing three-day-old SPF chicks, blood was drawn every seven days four consecutive times. The immunized chickens were challenged with virulent virus strains at 28 dpi and continuously observed for seven days; (**B**) SPF chickens were immunized with rDHN3-mF, rAd5-F, and rAd5-VP2-F2A-F, respectively. The PBS and rAd5-EGFP groups were used as controls. The antibody titers exceeded ≥1159 and were considered positive for the induction of anti-NDV response; (**C**) The SPF chickens were immunized with commercial HVT-VP2 vector vaccines, rAd5-VP2, and rAd5-VP2-F2A-F, respectively. The PBS and rAd5-EGFP groups were used as controls. The antibody titers > 396.39 were considered positive for the induction of the anti-IBDV response (ns—non-significant; ** *p* < 0.01; *** *p* < 0.001; **** *p* < 0.0001).

The immunization and challenge of SPF chickens in each group were performed as depicted in Figure 3A. Serum samples collected from chickens in NDV groups at 7, 14, 21, and 28 dpi were detected by ELISA. At 21 dpi, all chickens in the rDHN3-mF group, four out of five in the rAd5-F group, and three out of five in the rAd5-VP2-F2A-F group tested positive (Figure 3B). At 28 dpi, all chickens in these three groups were positive, with the mean antibody titers significantly increased compared to those at 21 dpi (Figure 3B). Comparative analysis of their mean antibody titers demonstrated that significantly higher mean titers were found in the rDHN3-mF group than those in rAd5-F and rAd5-VP2-F2A-F groups (*p* < 0.0001) at both 21 and 28 dpi. However, no significant differences were found between the rAd5-F group and the rAd5-VP2-F2A-F group (*p* > 0.05) ((Figure 3B). Chickens had no antibody response in both PBS and rAd5-EGFP groups throughout the immunization period (Figure 3B).

Serum samples collected from chickens in IBDV groups tested negative in the first two weeks (Figure 3C). However, at 21 dpi, all SPF chickens in the HVT-VP2 vector vaccine group, the rAd5-VP2 group, and the rAd5-VP2-F2A-F group tested positive, and the antibody titer increased at 28 dpi (Figure 3C). Similarly, the antibody levels detected in both rAd5-VP2 and rAd5-VP2-F2A-F groups were significantly lower than that in the HVT-VP2 vaccine group (*p* < 0.001), and no significant difference was found between rAd5-VP2 and rAd5-VP2-F2A-F groups (*p* > 0.05) (Figure 3C). These results demonstrate that recombinant adenoviruses can stimulate the production of specific antibodies in chickens but at lower levels compared to antibody levels stimulated by the attenuated strain rDHN3-mF and the commercial HVT-VP2 vector vaccine.

### 3.4. Assessment of the Cellular Immune Response

**Figure 4:**
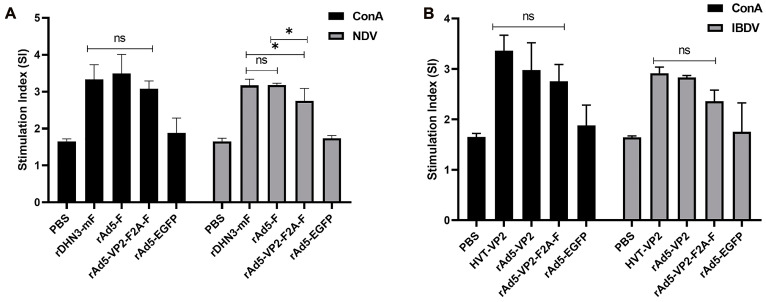

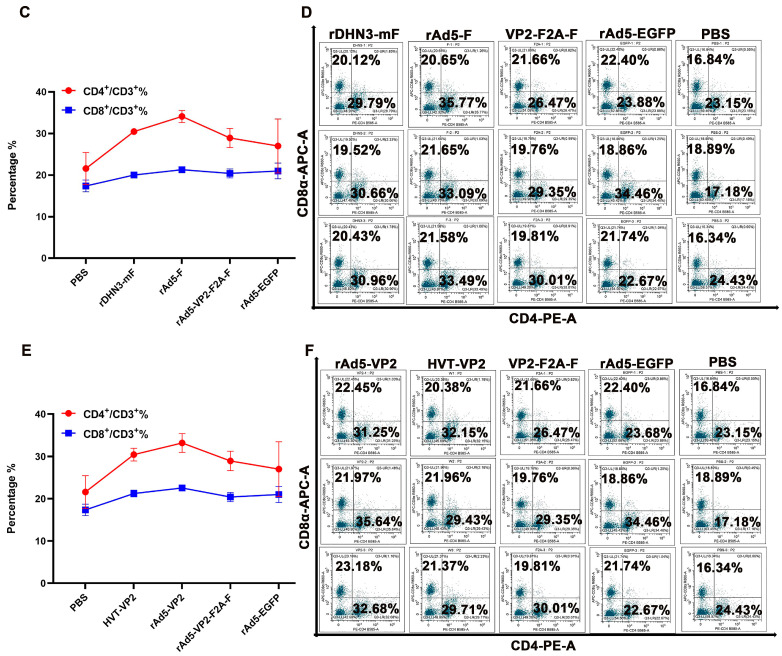
Comparison of cellular immunity in different groups: (**A**) Statistical analysis of the peripheral blood lymphocyte stimulation index in the ND groups. Under ConA stimulation, the SI values of peripheral blood lymphocytes in the PBS group were significantly lower than those of the rDHN3-mF, rAd5-F, and rAd5-VP2-F2A-F groups (*p* < 0.001). The SI values of peripheral blood lymphocytes in the rAd5-EGFP group were significantly lower than those in the rDHN3-mF, rAd5-F, and rAd5-VP2-F2A-F groups (*p* < 0.01). However, there was no significant difference between the rDHN3-mF, rAd5-F, and rAd5-VP2-F2A-F groups (*p* > 0.05), and no significant differences between the PBS and rAd5-EGFP groups (*p* > 0.05). Under inactivated NDV stimulation, the SI values of peripheral blood lymphocytes in the PBS and rAd5-EGFP groups were significantly lower than those in the rDHN3-mF, rAd5-F, and rAd5-VP2-F2A-F groups (*p* < 0.0001). SI values in the rAd-VP2-F2A-F group were significantly different from those of the rDHN3-mF and rAd5-F groups (*p* < 0.05); no significant differences between the rDHN3-mF and rAd5-F groups (*p* > 0.05), and there was no significant difference between the PBS and rAd5-EGFP groups (*p* > 0.05) (ns—non-significant; * *p* < 0.05); (**B**) Statistical analysis of the peripheral blood lymphocyte stimulation index in IBD groups. From (**D**) above, it can be seen that the SI values of peripheral blood lymphocytes in the HVT-VP2 vector vaccines group, rAd5-VP2 group, and rAd5-VP2-F2A-F group were not significantly different from each other regardless of ConA stimulation or inactivated IBDV stimulation in the IBD group (*p* > 0.05). However, the SI values of the above three groups were significantly higher than those of the rAd5- EGFP and the PBS groups (*p* < 0.05). There was no significant difference between the PBS and rAd5-EGFP groups (*p* > 0.05) (ns—non-significant); (**C**,**D**) Statistical analysis of the percentage of CD4+ and CD8+ T lymphocytes in the peripheral blood in ND groups. It can be seen from these two pictures that the percentages of CD4+ and CD8+ T lymphocytes in the rAd5-F, rDHN3-mF, and rAd5-VP2-F2A-F groups were significantly higher than those in the PBS group (*p* < 0.05). There was no significant difference between the above three vaccine groups (*p* > 0.05); (**E**,**F**) Statistical analysis of the percentage of CD4+ and CD8+ T lymphocytes in the peripheral blood in the IBD groups. It can be seen from these two pictures that the percentage of CD4+ and CD8+ T lymphocytes in the rAd5-VP2 group, the HVT-VP2 vector vaccines, and rAd5-VP2-F2A-F groups were significantly higher than in the PBS group (*p* < 0.05). There was no significant difference between the three vaccine groups (*p* > 0.05).

Peripheral blood lymphocyte proliferation ratios were first used to evaluate cellular immunity induced by immunization with the recombinant adenoviruses. Under ConA stimulation, the SI values of peripheral blood lymphocytes in PBS and rAd5-EGFP groups were significantly lower than those in the rDHN3-mF, rAd5-F, and rAd5-VP2-F2A-F groups (*p* < 0.001, *p* < 0.01). Still, there was no significant difference between rDHN3-mF, rAd5-F, and rAd5-VP2-F2A-F groups (*p* > 0.05) and between PBS and rAd5-EGFP groups (*p* > 0.05) (Figure 4A). However, under the stimulation with inactivated NDV, the SI values of peripheral blood lymphocytes in PBS and rAd5-EGFP groups were significantly lower than those in rDHN3-mF, rAd5-F, and rAd5-VP2-F2A-F groups (*p* < 0.0001); the SI values in rAd-VP2-F2A-F group were significantly lower than those in rDHN3-mF and rAd5-F groups (*p* < 0.05); and there was no significant difference between the rDHN3-mF and rAd5-F groups (*p* > 0.05), and between PBS and rAd5-EGFP groups (*p* > 0.05) (Figure 4A). These data indicated that immunization of SPF chickens with rAd5-F and rAd5-VP2-F2A-F effectively elicited the cellular immune response.

The SI values of peripheral blood lymphocytes in the HVT-VP2, rAd5-VP2, and rAd5-VP2-F2A-F groups were not significantly different from each other regardless of stimulation with ConA or inactivated IBDV (*p* > 0.05). Still, they were significantly higher than those of the rAd5- EGFP and PBS groups (*p* < 0.05) (Figure 4B). There was no significant difference between the PBS and rAd5-EGFP groups (*p* > 0.05) (Figure 4B).

The percentage of CD4+ and CD8+ T lymphocytes in peripheral blood was then analyzed to directly assess the cellular immune response in the immunized birds [24]. The results showed that the percentages of CD4+ T and CD8+ T lymphocytes in the rAd5-F, rDHN3-mF, and rAd5-VP2-F2A-F groups were significantly higher than those in the PBS group (*p* < 0.05), but with no significant difference between the three vaccine groups (*p* > 0.05) (Figure 4C,D). Similarly, the percentages of CD4+ T lymphocytes and CD8+ T lymphocytes in the groups rAd5-VP2, HVT-VP2, and rAd5-VP2-F2A-F were significantly higher than in the PBS group (*p* < 0.05), but with no significant differences between the three vaccine groups (*p* > 0.05) (Figure 4E,F). These results indicated that the recombinant adenoviruses could induce efficient cellular immune responses in SPF chickens similar to the attenuated strain rDHN3-mF and the commercial HVT-VP2 vector vaccine. In addition, it was also observed that the response of CD4+ T lymphocytes was more potent than CD8+ T lymphocytes in SPF chickens in both the NDV and IBDV groups.

### 3.5. Protective Efficacies against DHN3 Challenge

**Figure 5:**
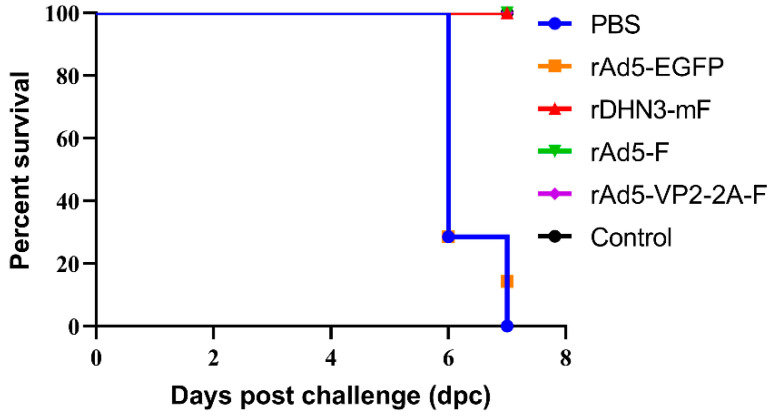
Survival curves of SPF chickens in ND groups after challenge with DHN3: Each chicken was injected with 10^5^ EID_50_/0.1mL virus. The survival rates of chickens in each group were recorded, and the survival curves were presented.

Chickens in NDV groups were challenged with the virulent NDV strain DHN3 at 28 dpi. From 3 dpc, SPF chickens in the PBS and rAd5-EGFP groups began to show typical clinical signs, such as decreased appetite, depression, lethargy, fluffy feathers, and row yellow-greenish dilute feces. In contrast, chickens in the rDHN3-mF, rAd5-F, and rAd5-VP2-F2A-F groups did not show these symptoms. Five SPF chickens each in the PBS and rAd5-EGFP group died at 6 dpc, the last two in the PBS group also died at 7 dpc, and one in the rAd5-EGFP group died at 7 dpc (Figure 5). The SPF chickens in the rDHN3-mF, rAd5-F, and rAd5-VP2-F2A-F groups survived, the same as in the control group (Figure 5).

Detection of the viral shedding of each SPF chicken in this group at 3, 5, and 7 dpc, respectively, showed no shedding of DHN3 in the rDHN3-mF group (Table 3). However, DHN3 shedding was detected in one SPF chicken each in both rAd5-F and rAd5-VP2-F2A-F groups at 7 dpc and in all the SPF chickens in rAd5-EGFP and PBS groups (Table 3). These results demonstrated that immunization of chicks with the recombinant adenoviruses rAd5-F and rAd5-VP2-F2A-F significantly reduced viral shedding but with slightly fewer efficacies compared to the NDV attenuated strain rDHN3-mF.

### 3.6. Protective Efficacies against BC6/85 Challenge

**Figure 6:**
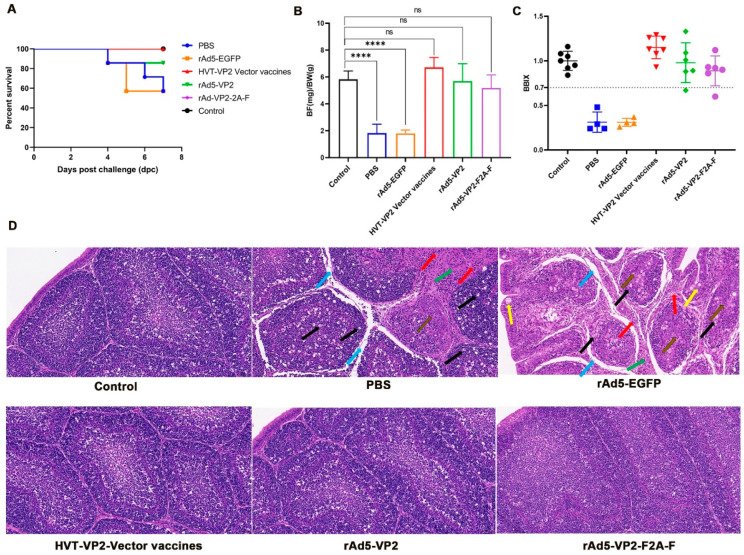
Survival curves and pathological changes in IBD groups after challenge with BC6/85: (**A**) Survival curves of SPF chickens in IBD groups after challenge with BC6/85. Each chicken was injected with 50 MID/0.2 mL virus, and the survival rates of the chickens in each group were recorded, and the survival curves were presented; (**B**) The ratio of the bursa of Fabricius (BF) to the body weight (BW). The mean BF/BW values of the PBS and rAd5-EGFP groups were significantly lower than those of the control group, as seen in the figure (*p* < 0.0001). However, the mean BF/BW values of the HVT-VP2 vector vaccines group, rAd5-VP2 group, and rAd5-VP2-F2A-F group were not significantly different from the control group (*p* > 0.05) (ns—non-significant; **** *p* < 0.0001); (**C**) Statistical analysis of the SPF chicken bursal index in IBD groups. This Figure shows that among the surviving SPF chickens, all SPF chickens in the PBS and rAd5-EGFP groups had bursa atrophy. In addition, one SPF chicken in each of the rAd5-VP2 and rAd5-VP2-F2A-F groups showed bursal atrophy, while no SPF chickens in both the HVT-VP2 vector vaccines and control groups showed bursal atrophy. The colors of the different groups in group (**C**) correspond to groups (**B**,**A**); (**D**) Bursal histopathological characteristics (H&E, 200×). Mild mucosal epithelial hyperplasia was indicated locally in the bursa of Fasciola as indicated with a yellow arrow; necrosis and dissolution in lymphoid follicles and medullary regions, pyknosis and deep staining or fragmentation of nuclei were indicated with a black arrow; undifferentiated epithelial hyperplasia at the junction of cortex and medulla were indicated with a brown arrow; extensive edema and widened space between lymphoid follicles were indicated with a blue arrow; and mild hyperplasia of follicular interstitial connective tissue was indicated with a green arrow; Scattered infiltration of inflammatory cells is indicated by a red arrow.

After the challenge with BC6/85, SPF chickens in PBS and rAd5-EGFP groups began to show clinical signs, including depression and loss of appetite at 3 dpc. One SPF chicken in the PBS group died each at 4, 6, and 7 dpc, one SPF chicken in the rAd5-EGFP group died at 4 dpc, and two at 5 dpc (Figure 6A). One SPF chicken, each in rAd5-VP2 and rAd5-VP2-F2A-F groups, died at 4 dpc, after which no further deaths occurred (Figure 6A). The HVT-VP2 group did not experience any mortality (Figure 6A). Dissection of the dead SPF chickens in the PBS and rAd5-EGFP groups revealed typical signs, such as hemorrhage in the leg and pectoral muscles, hemorrhage in the bursa of Fasciola, and enlarged kidneys.

The remaining survival SPF chickens were euthanized at 7 dpc and dissected. All surviving SPF chickens in PBS and rAd5-EGFP groups had bursa atrophy, one SPF chicken each in rAd5-VP2 and rAd5-VP2-F2A-F groups had bursa atrophy, and all SPF chickens in the HVT-VP2 vector vaccines group did not show the above symptoms (Figure 6B,C). Histopathological examination of the bursa of Fabricius of SPF chickens in the PBS and rAd5-EGFP groups showed extensive necrosis, dissolution of lymphoid follicles and cells in the medullary region, pyknosis and hyperchromatic or fragmented nuclei, extensive edema between lymphoid follicles, widened spaces, and scattered infiltration of more inflammatory cells (Figure 6D). A small amount of follicular cell necrosis in the rAd5-VP2 and rAd5-VP2-F2A-F groups was observed, with no other obvious abnormality (Figure 6D). No significant lesion was found in chickens in the HVT-VP2 group (Figure 6D). These results cumulatively demonstrated that the recombinant adenoviruses rAd5-VP2 and rAd5-VP2-F2A-F could protect the immunized chickens from atrophy of the bursa and other pathological damages but with slightly fewer efficacies compared to the commercial HVT-VP2 vector vaccine.

## 4. Discussion

NDV and IBDV are two important avian pathogens that have caused significant economic losses to the global poultry industry. The prevalent NDV strain in China is mainly genotype VII [25]. The very virulent IBDV (vvIBDV) that emerged in the 1990s with acute and highly lethal clinical signs was gradually replaced by novel variants, and atypical IBD continues to emerge [26,27]. Therefore, it is necessary to develop new vaccines against these two viral infections. In this study, we report the induction of efficient immune responses and protection against NDV and IBDV in chicks immunized with three recombinant adenoviruses expressing their major antigenic proteins, raising the possibility of developing these recombinant viruses as vaccine candidates. Furthermore, the observation that the combination and efficient expression of two antigens can be made in a single recombinant virus would also open a way to develop bi- and multivalent vaccines against these viral infections.

The main reasons for using the AdEasy system to construct recombinant adenovirus in this study are the following:

First, the commonly used attenuated ND or IBD vaccines are usually associated with a risk of re-infection. For example, when chickens immunized with an attenuated IBD vaccine with medium virulence induce a strong immune response, it often causes damage to the bursa of Fabricius and immune suppression due to the under-developed chicken immune system. The human adenovirus serotype 5 vector does not replicate in the animals, and the viral genome does not integrate into the host genome, thus avoiding the risk of spreading the virus and interfering with the expression of other host genes [28,29]. Second, in clinical practice, when chicks are immunized with attenuated or inactivated vaccines against ND or IBD, interference from maternal antibodies would often result in immunization failure. However, antibodies against human adenovirus are not usually present in poultry, such that no interference of maternal antibodies would occur [19]. Third, the system can be used in low doses and low production costs, with long immune protection time, short production cycles, and easy mass preparation [30,31]. Fourth, immune adjuvants were usually unnecessary, as the viral vector can function as a natural adjuvant and stimulate the innate immune system through both Toll-like receptor-dependent and independent pathways [32,33,34,35]. Fifth, immunization can be carried out in various ways, including the common intramuscular or subcutaneous injection, aerosol, or oral administration to elicit a local mucosal immune response [36]. Sixth, adenoviral vectors have the potential to insert a wide variety of exogenous genes, have a wide range of hosts, and are widely used in protein expression [17].

Adenoviral vectors have been widely investigated as potential vaccine candidates for the expression of animal virus proteins. Li et al. [37] cloned intron A, WPRE, CD40L, and GMCSF and the cap gene of porcine circovirus type 2 (PCV2) into the genome of an adenovirus and constructed the recombinant adenovirus Ad-A-CD40L-Cap-GMCSF-W, which can induce a more robust immune response than the commercial inactivated vaccine PCV2-SH strain. Hassan et al. [38] fused the relatively conserved immunogenic region [the M2 ectodomain (M2e), hemagglutinin (HA) fusion domain (HFD), T-cell epitope of nucleoprotein (TNP) and HA α-helix domain (HαD)] of H5N1 influenza virus to an adenoviral vector to construct a multi-epitope vaccine, and mouse experiments showed that this recombinant adenovirus provided good protection against H5, H7, and H9 subtypes of avian influenza. Tang et al. [39] used adenovirus as a vector to express the E protein of duck Tembusu virus (DTMUV). They constructed a recombinant adenovirus rAd-E with a final immune protection rate of 80% against DTMUV infection.

Other viral vectors, such as poxvirus [40], turkey herpes virus (HVT) [41], baculovirus [42], and infectious laryngotracheitis virus (ILTV) [43], have been used to express NDV antigens. Regardless of the platform used, increased immune protection in animals is usually found when both F and HN genes are co-expressed, compared to vaccines that express either the F or HN gene alone [44]. Therefore, it would be plausible that immunization of chickens with recombinant adenoviruses expressing both F and HN genes may boost the antibody response levels. However, not all SPF chickens immunized with rAd5-F and rAd5-VP2-F2A-F antibodies tested positive after three weeks of immunization. Antibody response levels were also lower than in those immunized with rDHN3-mF. One reason could be that the NDV ELISA kits used were coated with inactivated NDV. The attenuated strain rDHN3-mF stimulated chickens to develop anti-F protein antibodies and antibodies against other viral proteins. Determining and comparing specific antibody responses against F protein antibodies would help clarify this problem. This lower level of antibody response was also reflected in the challenge experiments. Although no mortality was observed in chickens immunized with either rAd5-F, rAd5-VP2-F2A-F, or rDHN3-mF, 14.29% of chickens immunized with rAd5-F or rAd5-VP2-F2A-F had viral shedding at 7 dpc. As immunization of chickens with rAd5-F and rAd5-VP2-F2A-F stimulated similar levels of the cellular immune response as rDHN3-mF, the weaker protection of viral shedding could be attributed to the weaker induction of antibody response by the two recombinant adenovirus vaccines. However, these results demonstrate the high effectiveness of recombinant adenoviruses rAd5-F and rAd5-VP2-F2A-F in preventing mortality but not viral shedding.

Similarly, SPF chickens immunized with rAd5-VP2 and rAd5-VP2-F2A-F produced lower antibody levels than commercial HVT-VP2 vector vaccines, possibly because HVT replicates in SPF chickens [45]. However, the replication-deficient HAdV-5 could not replicate in chickens after a transient infection. Therefore, the dose of the recombinant adenoviruses and the immunization times should be increased to enhance the antibody response. Furthermore, both recombinant adenoviruses stimulated levels of cellular immunity similar to those of the HVT-VP2 vector vaccine, confirming that the recombinant adenoviruses could stimulate efficient cellular immune responses. As the survival rate of chickens in both the rAd5-VP2 and rAd5-VP2-F2A-F groups was slightly lower than that in the HVT-VP2 group after the challenge, this incomplete protection may also be due to the lower antibody response in chickens immunized with the two recombinant adenoviruses.

In HEK293A cells infected with recombinant adenovirus rAd5-VP2-F2A-F, both VP2 and F proteins were efficiently expressed and detected. However, the full-length VP2-F2A-F protein was not detected, demonstrating the high efficiency of the (GSG) F2A cleavage peptide. This is consistent with previous studies [46,47]. Furthermore, immunization of chickens with this recombinant adenovirus revealed a similar protective efficacy as the recombinant adenovirus expressing a single gene. This high cleavage efficiency of the (GSG) F2A and excellent protection efficacy of rAd5-VP2-F2A-F would make the recombinant adenovirus a potential bivalent vaccine candidate against both NDV and IBDV infections.

## 5. Conclusions

In summary, the three recombinant adenoviruses, rAd5-F, rAd5-VP2, and rAd5-VP2-F2A-F, were manufactured using the AdEasy system in this study. After immunization, the three recombinant adenoviruses induced efficient humoral and cellular immunity in SPF chickens and provided partial protection against challenges with virulent strains. The findings of this study provide the initial evidence to support the further development of these three recombinant adenoviruses as vaccine candidates against NDV and IBDV infections in poultry.

## Figures and Tables

**Table 1:** Primer sequences and sizes of PCR fragments.

Primer	Sequence(5′–3′)	Fragment Length/bp
F-F	ATGGGCTCCAAACCTTCTACC	1662
F-R	TCATGCTCTCATGGTGGCTC	
VP2-F	ATGACAAACCTGCAAGATC	1356
VP2-R	CCTTATGGCCCGGATTATGTC	
H-F-F	**CGCTAGAGATCTGGTACCGTCGAC**GCCACCATGG GCTCCAAACCTTCTAC	1740
H-F-R	**CTTATCTAGAAGCTTAGGCTCGAG**TCACTTATCA TCGTCGTCCTTGTAGTCTGCTCTCATGGTGGCTCT	
H-VP2-F	**TCAGATCCGCTAGAGATCTGGTACCGTCGAC**GC CACCATGACAAACCTGCAAGATC	1424
H-VP2-R	**CGGATATCTTATCTAGAAGCTTAGGCTCGAG**CC TTATGGCCCGGATTATGTC	
P1	**TAGAGATCTGGTACCGTCGAC**GCCACCATGACA AAC	1415
P2	**GCCAACTTGAGCAGG**TCGAAGTTGCCGCTGCCC CTTATGGCCCGGATTATGTC	
P3	**CCTGCTCAAGTTGGC**CGGAGACGTTGAGTCCAA CCCTGGGCCCATGGGCTCCAAACC	1753
H-F-R	**CTTATCTAGAAGCTTAGGCTCGAG**TCACTTATCA TCGTCGTCCTTGTAGTCTGCTCTCATGGTGGCTCT	

Note: Homologous sequences are indicated in bold, and sequences for the flag tag in H-F-R primers and F2A in P2 and P3 primers are underlined (All primers were designed using SnapGene ^®^3.2.1 software).

**Table 2:** Grouping of SPF chicks and immunization procedures.

Isolator No.	Name of Immune Reagents	Immunization Dose (per Chick)	Number of SPF Chicks
1	HVT-VP2 vector vaccines	0.2 mL	7
2	rAd5-VP2	10^8^ IFU/0.2 mL	7
3	rAd5-VP2-F2A-F	10^8^ IFU/0.2 mL	14
4	rDHN3-mF	10^6^ EID_50_	7
5	rAd5-F	10^8^ IFU/0.2 mL	7
6	rAd5-EGFP	10^8^ IFU/0.2 mL	14
7	PBS	0.2 mL	14
8	Control	Not immune to any reagent	14

**Table 3:** Viral shedding detected by HA from trachea and cloaca swabs.

Group	3 dpc		5 dpc		7 dpc	
	Oropharynx	Cloaca	Oropharynx	Cloaca	Oropharynx	Cloaca
Control	0/7	0/7	0/7	0/7	0/7	0/7
rDHN3-mF	0/7	0/7	0/7	0/7	0/7	0/7
rAd5-F	2/7	1/7	1/7	1/7	1/7	1/7
rAd5-VP2-F2A-F	2/7	2/7	2/7	1/7	1/7	1/7
rAd5-EGFP	7/7	7/7	7/7	7/7	1/1	1/1
PBS	7/7	7/7	7/7	7/7	NA	NA

Note: NA: not available.

## Data Availability

Data is available on request from the authors.
